# Novel autopsy and genetic findings in an acardiac twin: case report and literature review

**DOI:** 10.4322/acr.2024.477

**Published:** 2024-03-05

**Authors:** Natalie Fabrizio, Christopher L. Pankey, Kathleen Martin, Michael Baker, Cameron Clark Felty

**Affiliations:** 1 West Virginia School of Osteopathic Medicine, Lewisburg, WV, United States; 2 Dartmouth-Hitchcock Medical Center, Department of Pathology and Laboratory Medicine, Lebanon, NH, United States

**Keywords:** Fetofetal Transfusion, Cloaca, Twins Monozygotic, genetics, Chromosomal Aberrations

## Abstract

Twin reversed arterial perfusion (TRAP) sequence is a rare complication of monochorionic twinning whereby a donor twin perfuses an acardiac twin via aberrant vascular anastomoses. The resulting paradoxical retrograde blood flow supplying the acardiac twin is oxygen-poor, leading to some of the most severe malformations encountered in humans. Though the first descriptions of acardiac twins date back to at least the 16^th^ century, the pathophysiologic processes which underpin the development of TRAP sequence are still being elucidated. Theories on the pathogenesis of TRAP sequence include deficiencies intrinsic to the embryo and primary abnormalities of the placental vasculature. Autopsy studies continue to provide clues to the underlying pathogenesis of TRAP sequence, and the characterization of the spectrum of manifestations that can be observed in acardiac twins. Herein, we present the clinical, autopsy, and molecular findings in a unique case of TRAP sequence. Novel findings include a primitive cloaca-like structure and chromosomal aberrations involving 6q11.1 and 15q25.1.

## INTRODUCTION 

Twin reversed arterial perfusion (TRAP) sequence is a severe manifestation of twin-twin transfusion syndrome (TTTS) whereby a donor twin perfuses an acardiac twin.^1^ While the condition is rare, the literature contains a broad range of estimates on the proportion of births affected by TRAP sequence. Based on historical data, an estimate, commonly provided in the literature asserts that the condition affects 1 in 35,000 births.^2,3^ However, a more recent study by Botto et al.^4^ suggests that this commonly provided figure is likely overestimated, with the total prevalence being closer to 1 in 70,000 births. The condition is observed in monochorionic multifetal gestations, commonly twins, though cases involving triplet, quadruplet, and quintuplet gestations have been reported.^5-10^ While most cases occur in monozygotic twins, dizygotic twins can also be affected.^11^ Reports of acardia date back to the 16^th^ century, though nebulous descriptions of headless peoples with their eyes and mouth attached to their chest date back even further.^12^ It wasn’t until the 18^th^ century that acardia was associated with twin gestations, and much progress has been made in unraveling the mysteries surrounding TRAP sequence since that time.^12^

In normal fetal circulation, the placenta supplies the growing fetus with oxygen and nutrients while facilitating the removal of waste products. Oxygenated blood from the placenta is transported to the fetus via the umbilical vein, which feeds into the fetal hepatic circulation and inferior vena cava. After passing through the heart and systemic circulation, deoxygenated blood is returned to the placenta via two umbilical arteries.^13^ In normal twin gestations, separate umbilical cords coordinate the blood flow of each twin independently.^14^ TRAP sequence occurs in monochorionic multifetal gestations where direct umbilical arterioarterial (A-A) and venovenous (V-V) anastomosis form between the donor twin and acardiac twin. In addition, the acardiac twin either completely lacks or possesses only rudimentary non-functional heart tissue. As a result of these factors, blood flows in a retrograde manner from the umbilical artery of the donor twin to the umbilical artery of the acardiac twin via the intertwin vascular anastomoses. The acardiac twin is thus only perfused with shunted hypoxic blood supplied by the cardiac output from the donor twin.^15^ The abnormal circulation not only precipitates malformations in the acardiac twin but also poses a risk to the donor twin. While TRAP sequence is uniformly lethal for the acardiac twin, the mortality rate in the donor twin is estimated at 30-50% and is principally related to heart failure.^4^ Considering that hemodynamics are a primary contributor to cardiac development in utero, the increased cardiac demand required for the donor twin to act as the “pump” for the acardiac twin drives the increased risk of cardiovascular disease seen post-utero.^16^

One hypothesis on the pathogenesis of the TRAP sequence posits that the condition is related to a primary deficiency in the embryo. Proposed mechanisms leading to the embryonic defect include unequal splitting of cells during the initiation of the monozygotic twinning process, chromosomal aberrations, and epigenetic modifications. The resulting embryonic deficiency is intrinsically fatal for the acardiac twin, and the presence of abnormal placental vascular anastomoses permits its continued development.^4,5,17,18^ Alternative hypotheses postulate that the formation of aberrant vascular anastomosis is the primary pathogenic event. In this context, the subsequent perfusion of the acardiac twin with oxygen and nutrient-poor blood leads to lethal injury and malformations.^4,5^ The latter hypothesis is favored in the modern literature and led to the referral of acardiac twinning as TRAP sequence, reflecting the suspected pathogenic mechanism.^5^

Though much progress has been made in unraveling the mysteries surrounding TRAP sequence, case reports with novel findings continue to be added to the literature. Additionally, autopsy studies provide unique opportunities to further refine our understanding of the morphologic manifestations of TRAP sequence and its underlying pathogenesis. Herein, we present an autopsy case of an acardiac twin with novel genetic and morphologic findings.

## CASE REPORT

A 27-year-old gravida 7, para 2, woman presented to the hospital in pre-term labor at 26 weeks gestation. Her obstetric history was complex, including a prior twin pregnancy with intrauterine demise of one twin at 13 weeks gestation, prior molar pregnancy, and a child with congenital 22q11 deletion syndrome. A morphology scan performed upon arrival revealed a monochorionic twin pregnancy complicated by TRAP sequence and marked polyhydramnios. On hospital day 2, an amnioreduction was performed for patient comfort, with 2.4 liters of amniotic fluid removed. On hospital day 3, the mother developed severe, unrelenting abdominal pain and was noted to be 6 cm dilated. An emergent primary low transverse cesarean section was performed for presumed placental abruption. A presenting non-viable acardiac twin was delivered first, followed by a viable female infant. The postpartum course for both the mother and viable infant was without complication.

### Autopsy findings

**Figure 1:**
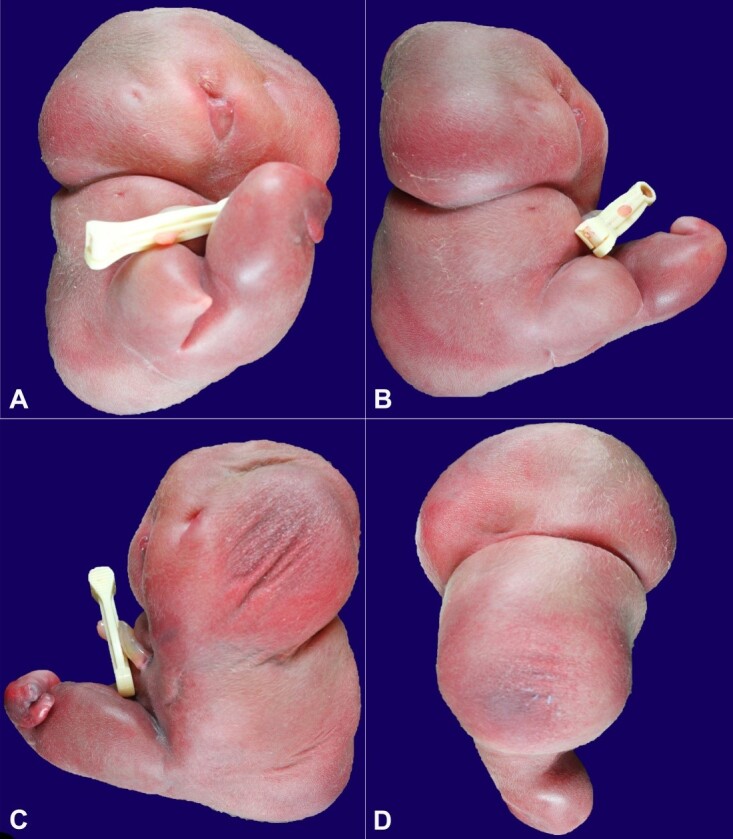
External examination of the acardiac twin with severe fetal hydrops. **A -** Anterior view showing lack of mature midline facial structures with blind-ended primitive oral cavity; **B -** Right lateral view demonstrating complete absence of right upper extremity and distal right lower extremity; **C -** Left lateral view demonstrating complete absence of left upper extremity and rudimentary left lower extremity; **D -** Posterior view demonstrating lack of rectoanal structures.

The acardiac twin weighed 559 grams, corresponding to <5^th^ percentile for gestational age. On external examination, severe fetal hydrops was present (Figure 1).

**Figure 2:**
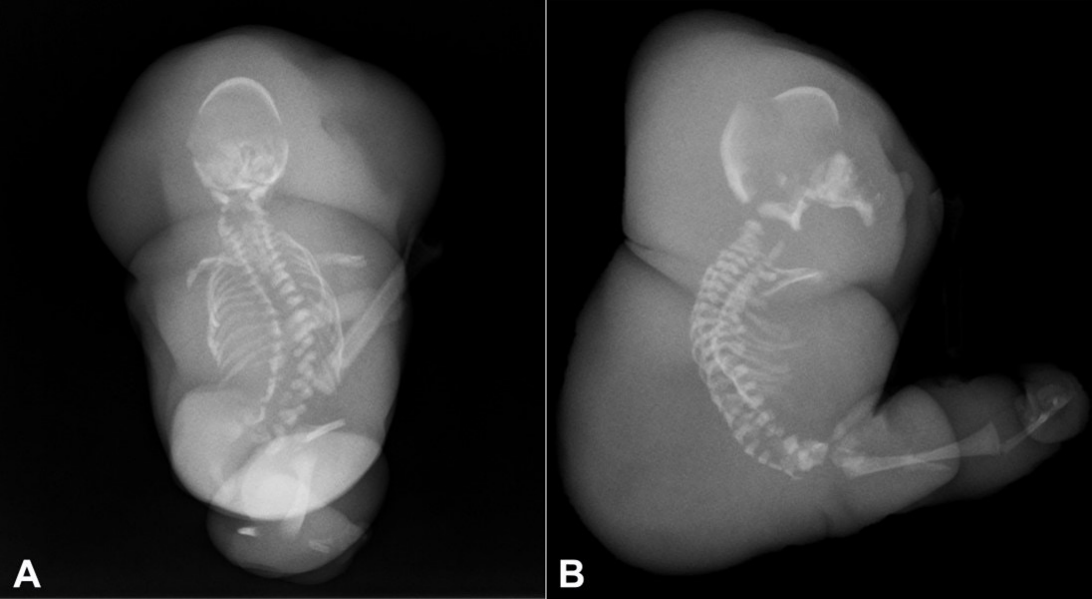
Postmortem Xray. **A -** AP view; **B -** lateral view demonstrating hypoplasia of the appendicular skeleton, small, malformed cranium, and thoracolumbar scoliosis with multiple vertebral anomalies.

The head was poorly developed and without mature midline facial structures. A primitive, blind-ended oral cavity with a small, retracted tongue was present. No mature eyes or ears were identified. The trunk was poorly developed, and no mature external genitourinary or rectoanal structures were present. The upper extremities were completely absent. The lower extremities were anomalous and rudimentary, with the lower leg and foot completely absent on the right. The left foot consisted of two metatarsals and two digits. Post-mortem X-ray confirmed the gross findings of severe fetal hydrops and hypoplasia of the appendicular skeleton (Figure 2).

Additionally, the structures of the axial skeleton were anomalous with a relatively small, malformed cranium, thoracolumbar scoliosis, and multiple other non-classifiable vertebral abnormalities.

**Figure 3:**
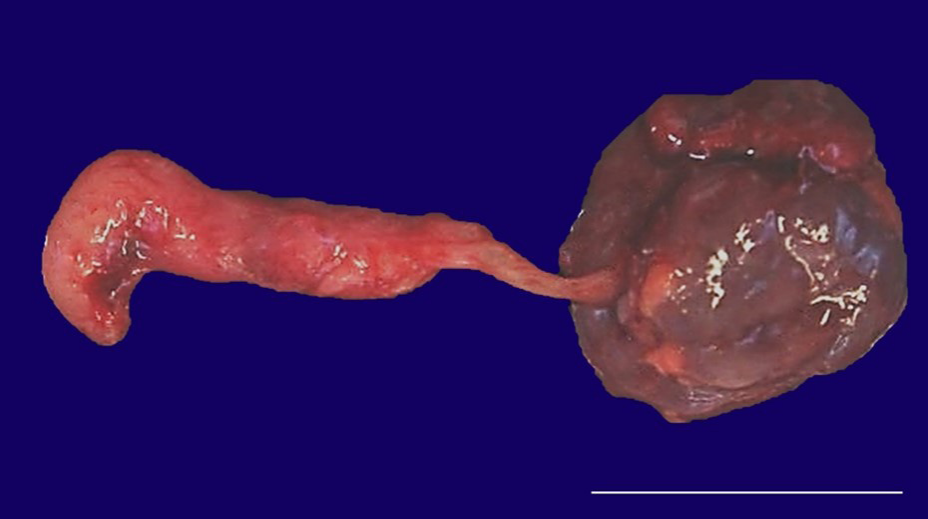
Gross appearance of anomalous cloaca-like structure consisting of a tubular structure which was contiguous with the renal collecting system (scale bar= 2.0 cm).

On internal examination, generalized soft tissue edema was noted. Cystically dilated lymphatic malformations were identified within the soft tissues of the head, trunk and lower extremities. The thoracoabdominal cavities contained an incomplete compliment of internal organs. One globoid kidney and adrenal gland were grossly identified within the abdominal cavity. Microscopic examination of the kidney and adrenal gland revealed age-appropriate development. Interestingly, an anomalous tubular structure with a central lumen and smooth muscle wall was contiguous with the renal collecting system (Figure 3).

**Figure 4:**
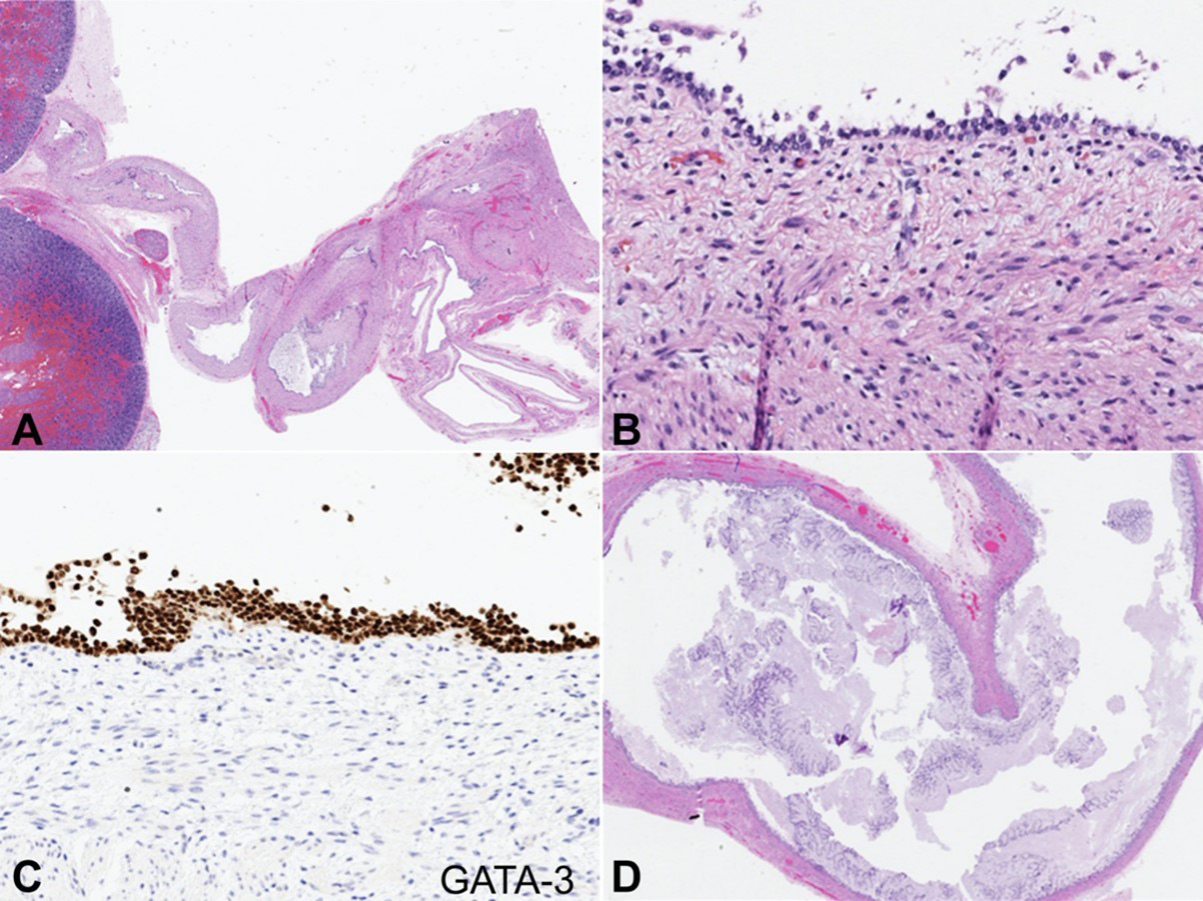
Microscopic appearance of anomalous cloaca-like structure. **A -** The cloaca-like structure was contiguous with the renal collecting system (H&E stain, 10x); **B -** The proximal segment was lined by urothelium (H&E stain, 380x); **C -** The proximal segment demonstrated strong GATA-3 expression by immunohistochemistry (360x); **D -** The mid-segment was lined by intestinal epithelium (H&E stain, 25x).

**Figure 5:**
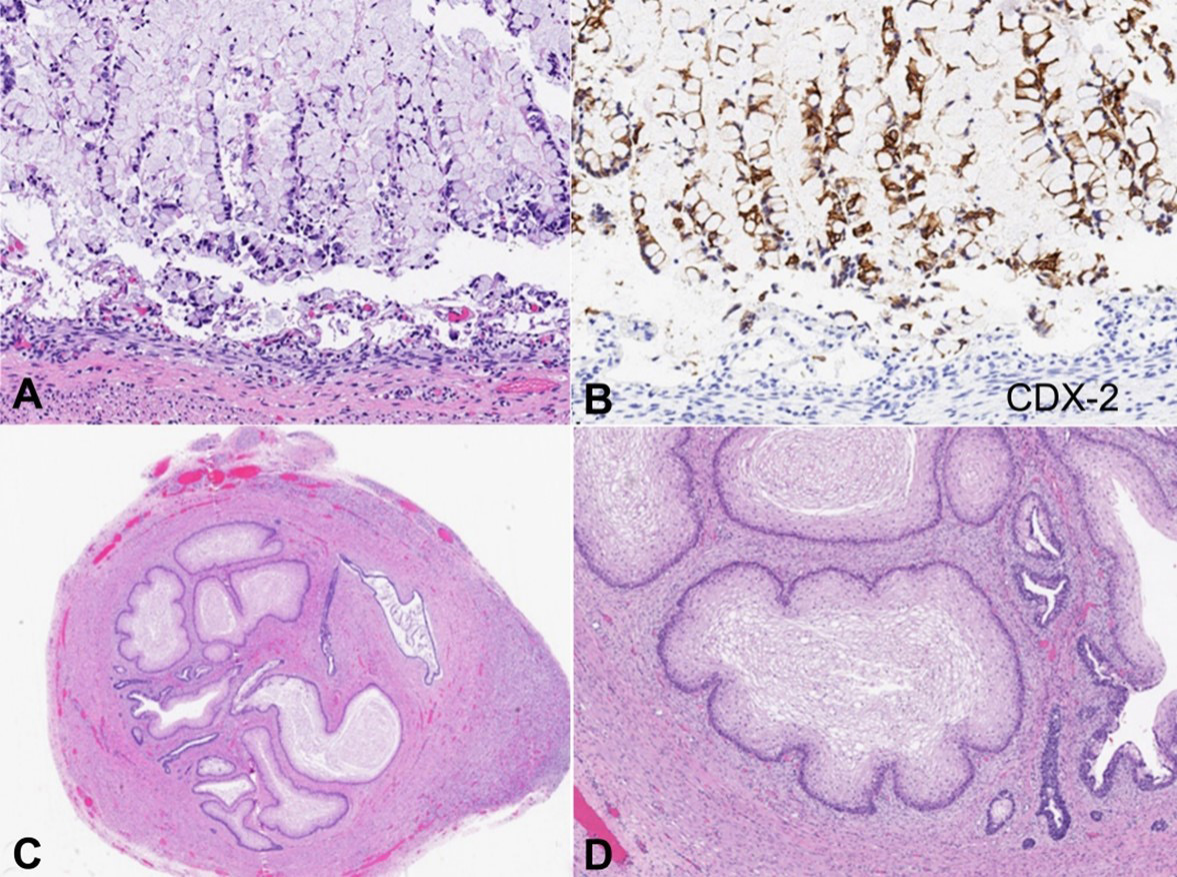
- Microscopic appearance of anomalous cloaca-like structure. **A -** The mid-segment was lined by intestinal epithelium (H&E stain, 250x); **B -** The mid-segment demonstrated strong CDX-2 expression by immunohistochemistry (250x); **C** and **D -** The distal segment was lined by stratified squamous epithelium (H&E stain, 40x and 200x).

**Figure 6:**
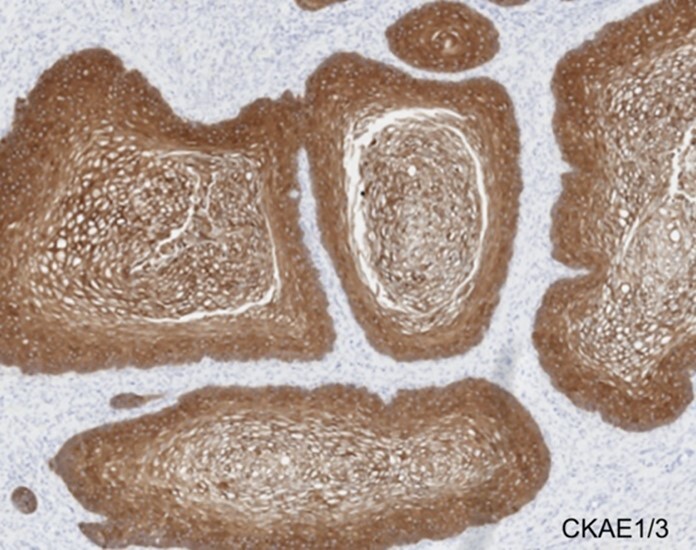
Microscopic appearance of anomalous cloaca-like structure. The stratified squamous epithelium demonstrated only strong CKAE1/3 expression by immunohistochemistry (200x).

The proximal segment was lined by urothelium, the mid-segment by intestinal epithelium, and the distal segment by squamous epithelium, each confirmed by immunohistochemical markers of differentiation (Figure 4, 5 and 6).

Taken together, these findings are most indicative of a primitive cloaca-like structure. Rudimentary lung, pancreatic and ovarian tissues were also identified microscopically. No additional thoracoabdominal organs were identified either grossly or microscopically.

**Figure 7:**
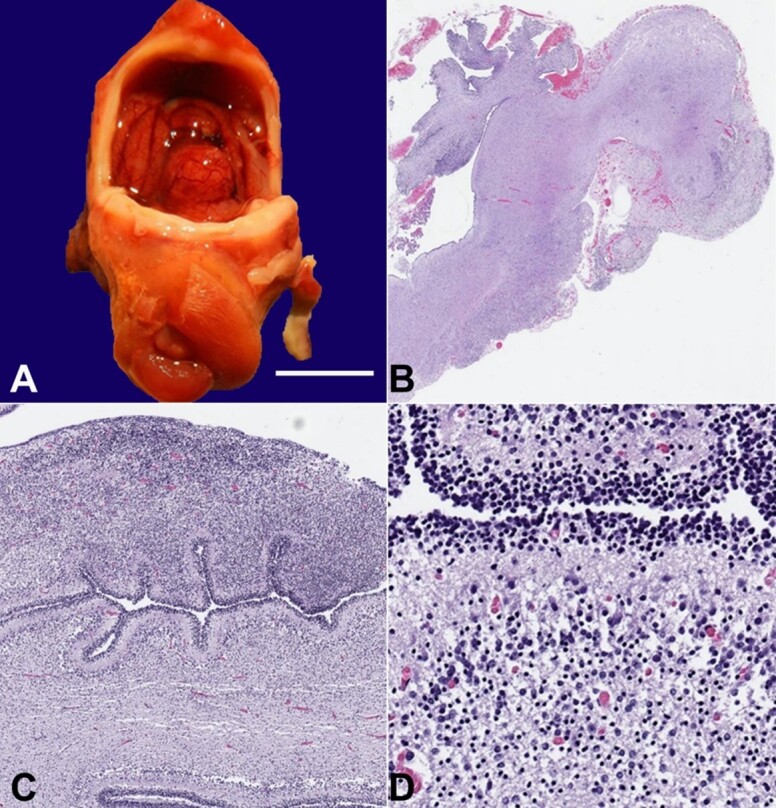
Neuropathology. **A -** In-situ examination of the brain revealed a rudimentary brainstem, hypoplastic cerebellum, and complete absence of the cortical hemispheres (scale bar= 1.0 cm); **B -** Whole mount of the rudimentary brainstem with hypoplastic cerebellum (H&E stain, 20x); **C** and **D -** Immature cerebellum with external granular, molecular, Purkinje, and internal granular layers (H&E stains, 230x and 350x).

Gross and microscopic examination of the brain revealed a rudimentary brainstem and cerebellum with complete absence of the cortical hemispheres (Figures 7A and 7B). The cerebellum was poorly-developed with recognizable external granular, molecular, Purkinje, and internal granular layers (Figures 7C and 7D).

A possible rudimentary olivary nucleus was identified microscopically in the area of the presumed medulla. The remaining areas consisted of a mixture of immature neuronal elements, immature glial elements, mature neurons and ependymal rests.

**Figure 8:**
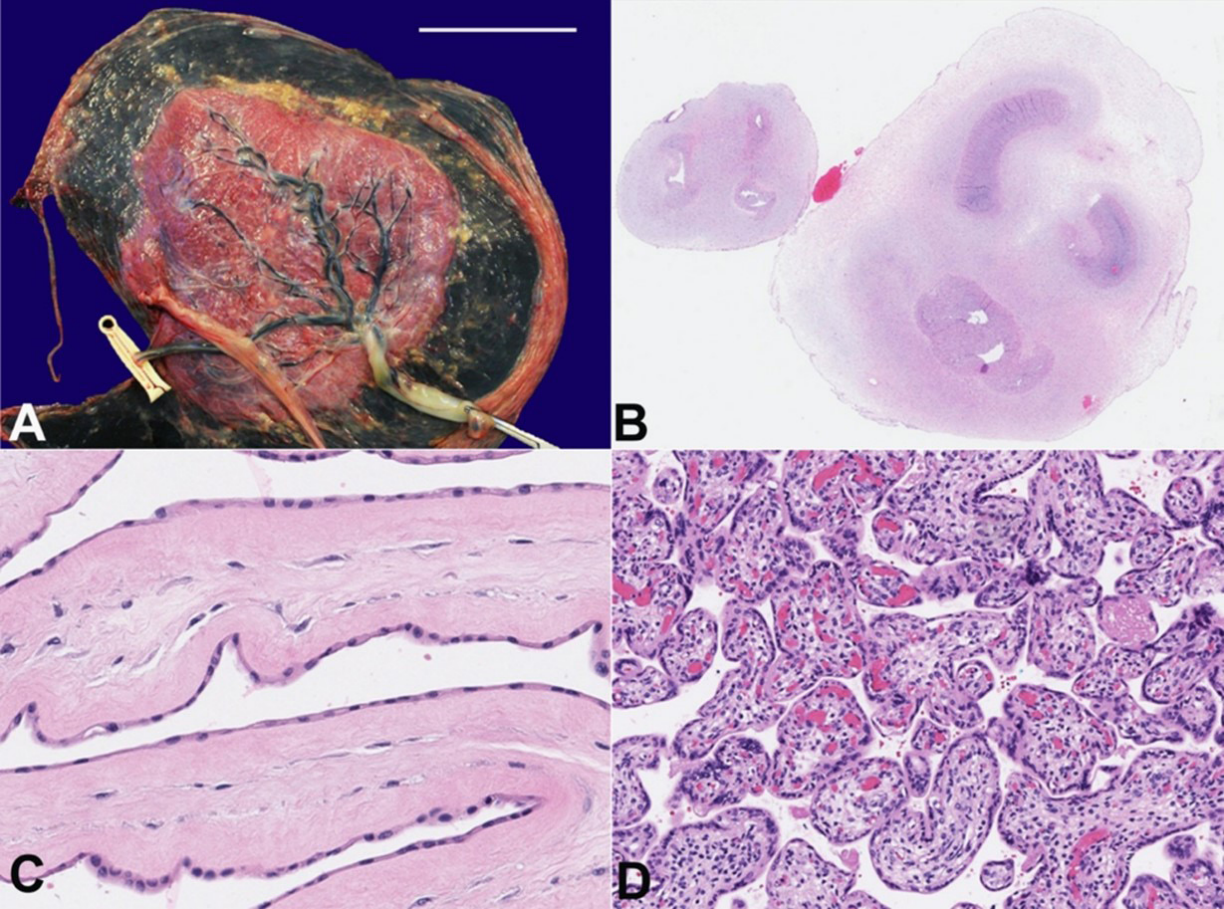
Placenta. **A -** Gross appearance of the twin gestation placenta with two fetal sacs (scale bar= 8.5 cm); **B -** Microscopic appearance of the 3-vessel umbilical cords belonging to the acardiac (smaller cord) and donor (larger cord) twins (H&E stain, 25x); **C -** Monochorionic diamniotic membranes (H&E stain, 400x); **D -** Developmentally appropriate placental villi (H&E stain, 180x).

The weight of the twin gestation placenta corresponded to <10th percentile of that expected for gestational age (Figure 8A). The fetal sac occupied by the donor twin accounted for 90% of the fetal surface, and the sac occupied by the acardiac twin accounted for the remaining 10%. The umbilical cord belonging to the acardiac twin was velamentous while the cord belonging to the donor twin was eccentrically inserted. The umbilical cords were of unequal length and caliber, with the smaller belonging to the acardiac twin. Each umbilical cord contained three vessels (Figure 8B). A direct umbilical arterial anastomosis traversing the dividing membrane was identified by gross examination aided by intravascular ink injection. Microscopic examination revealed developmentally appropriate placental villi and monochorionic, diamniotic membranes (Figure 8C and 8D).

A Cytoscan high density (HD) single nucleotide polymorphism (SNP) array, performed on DNA isolated from tissues harvested from the acardiac twin, revealed a female karyotype. Interestingly, two copy number aberrations were detected: copy number loss (x1) of 6q11.1 and copy number gain (x3) of 15q25.1.

## DISCUSSION

A classification system presented by Das in 1902 is still commonly used in modern literature, which subcategorizes acardiac twins into 4 groups based on morphologic features (Table 1).^19,20^

**Table 1:** Morphologic classification of acardiac twins with relative frequencies

**Subtype**	**Morphologic Features**	**Frequency (%)**
Acardiac acephalic	Pelvis and lower extremities are present without development of cranio-thoracic and upper extremity structures	60-75​
Acardiac anceps	Trunk and extremities are developed with partial development of cranial structures	20​
Acardiac acormus ​	Development of cranial structures only	10​
Acardiac amorphous ​	Amorphous tissue mass​	5​

Given the spectrum of malformations observed, our case is best classified as acardiac anceps. This rare case of acardiac-anceps is made even more interesting by the presence of a cloaca-like structure and the identification of novel chromosomal aberrations.

The cloaca is an embryonic structure that serves as a terminal channel shared by the digestive and urogenital tracts. The structure develops by the 4^th^ week and quickly becomes partitioned into the rectum and urogenital sinus. The partitioning occurs as the urorectal septum grows toward the cloacal membrane and lateral mesodermal ridges extend. The cloaca becomes divided into the rectum and urogenital sinus by approximately 6-7 weeks.^21,22^ The epithelium lining the tubular structure identified in our case consisted of intestinal, urothelial, and non-keratinizing squamous elements. The epithelial elements are consistent with the expected components of both the intestinal and genitourinary tracts, suggesting that structure is a cloaca derivative arrested around the 4^th^-5^th^ week of development. Lewitowicz et al.^23^ also reported the finding of a cloaca-like structure in an acardiac acephalic twin. In this case, histologic examination of the intestine revealed colonic epithelium with foci of urothelium forming a “cloacal-like space”. To our knowledge, this is the only additional report documenting the presence of a cloaca-like structure in an acardiac twin.

Chromosomal abnormalities are reportedly present in up to 33% of acardiac twins.^24^ The review of the cytogenetic aberrations identified in acardiac twins revealed a broad array of abnormalities (Table 2).^4,8,9,10,25-40^

**Table 2:** Genetic anomalies identified in acardiac twins

**Ref**	**Acardiac Twin**			**Pump Twin**		
	**Result**	**Tissue**	^#^ **Cells**	**Result**	**Tissue**	**# Cells**
^25^	46,XY/47,XY,+C	F	9/38	46, XY	L	-
^26^	46, XX/47,XX,+ minute chromosomal fragment	F	27/4	46,XX/47,XX + minute chromosomal fragment	L	27/1
				46,XX	F	9
^26^	46,XY	L	40	46,XY	L	38
	47,XY,+G	F	50	46,XY	F	40
^27^	47,XXY	A	-	47,XXY	A	-
		F			F	
^28^	45,X	F	20	46,XX	L	100
					F	20
^29^	70, XXX+15/see Note 1	F	22/9	46, XY	L	-
		F	7/21			
^30^	45, XX,t(4,21)del(4p)	U	-	46,XX	U	-
^31^	46,XX/47,XX,+11	F	-	46,XX	U	-
^32^	94,XXXXYY	U	-	47,XXY	U	-
^33^	Case 1: 94,XXXXYY	F	20	47,XXY	L	50
		L	20			
	Case 2: 47,XXY	F	20	47,XXY	L	18
^34^	46,X,i(Xp)	F	-	46, XX	L	-
^35^	46,XX,inv(10)(p12q25)	U	-	46,XX,inv(10)(p12q25)	U	-
^9^	46,XX/hypodiploidy/hyperdiploidy	L	18/10/2	46, XX (both co-triplets)	L	-
	46,XX	F				
^36^	48,XY,+12,+17	A	14	46,XY		
^8^	47,XY,+2	F	-	46,XY (pump co-triplet)	U	
^37^	47,XX,+2	F	-	46,XX	L	-
^38^	47,XX,+2	F	-	46,XX	U	20
^4^	47,XX,+13	-	-	U	-	-
^10^	47,XY,+ mar 15	-	-	U (both co-triplets)	-	-
^39^	Loss of heterozygosity at CSF1PO locus	Note 2	-	NP		
^40^	Trisomy 19	Note 3	-	46,XY/47,XY,+21	L	19/3
IC	XX with copy number loss (x1) of 6q11.1 and copy number gain (x3) of 15q25.1	F	-	NP	-	-

# = number; A = amniotic fluid cells; F = fibroblasts; L = lymphocytes; NP = not performed; Ref= reference; U = unknown specimen type; IC = index case. Note 1 - Random chromosome losses were seen in metaphase spreads with <70 chromosomes. Note 2 - Results from a DNA genotyping analysis by multiplex PCR at 16 short tandem repeat foci led a placental mass to be characterized as an acardiac fetus. Loss of heterozygosity at the CSF1PO locus was detected by multiplex PCR at 16 STR loci in 1 of 2 tested tissue samples from the acardiac fetus. Note 3 - Per the authors, a placental mass initially characterized as a teratoma was likely an acardiac fetus based on molecular results. Trisomy 19 was detected by STR(GenePrint24) analysis in tissue from the acardiac fetus.

To our knowledge, we are the first to report copy number loss of 6q11.1 and copy number gain of 15q25.1 in an acardiac twin. ​Copy number gains involving the distal region of 15q are rare and manifest with a wide range of growth, metabolic, neurodevelopmental, hematologic, cardiovascular, urogenital, and skeletal abnormalities. The features described in individual cases are sometimes opposing, such as overgrowth and growth restriction.^41-44^ Given the aforementioned range of abnormalities, it is not surprising that a number of morbid genes are present at 15q25.1, with an associated broad array of functions and disease correlates.^41^ Copy number losses involving the proximal region of 6q are also rare. A review of 12 cases by Hopkin et al.^45^ found that deletions involving 6q11 - 6q16 are commonly associated with hernias, upslanting palpebral fissures, and thin lips. Less common manifestations included foot anomalies, tracheoesophageal fistula, microcephaly, micrognathia, and cardiac anomalies. Engwerda et al.^46^ reviewed 11 cases of proximal 6q deletions involving 6q11q13, which generally showed similar findings aside from a lack of cardiac anomalies. Additional common dysmorphisms noted in that study included ear anomalies, joint hypermobility, genitourinary anomalies, and vertebral abnormalities.

While identifying novel copy number variants is certainly interesting, their contribution to the development of TRAP sequence in our case cannot be definitively stated. However, our case adds to the growing number of chromosomal anomalies associated with acardia. No consistent chromosomal aberrations have been identified in acardiac twins thus far, but this may be related to the rarity of TRAP sequence and inconsistency in obtaining genetic testing. Though the formation of placental vascular anastomosis is a pathogenic mechanism favored in the modern literature, it is reasonable to postulate that chromosomal aberrations may play a role in TRAP sequence, even if only in a subset of cases.

## CONCLUSION

While we have certainly become more nuanced in our understanding of TRAP sequence since the early historical accounts, each newly identified case offers opportunities to refine our understanding further. The salience of this paradigm is underscored by the novel findings identified in our case. Our case also highlights the pivotal role autopsy studies continue to play in investigating this intriguing phenomenon.
